# Biomedical applications and perspectives of two-dimensional porphyrin-based MOFs

**DOI:** 10.3389/fphar.2026.1819283

**Published:** 2026-04-20

**Authors:** Cheng Qi, Xuanxuan Luo, Huoying Pan, Weiqi Wang, Xiaohua Zheng

**Affiliations:** 1 The People’s Hospital of Danyang, Affiliated Danyang Hospital of Nantong University, Danyang, Jiangsu, China; 2 School of Pharmacy, Nantong University, Nantong, Jiangsu, China

**Keywords:** antibacterial treatment, biosensing, cancer therapy, metal-organic frameworks, porphyrin

## Abstract

Two-dimensional porphyrin-based metal-organic frameworks (2D Por-MOFs) have emerged as promising candidates in biomedical applications due to their ultrathin morphology, high surface area, tunable electronic properties, and excellent optical characteristics. This review systematically summarizes recent advances in their utilization for cancer therapy, antibacterial treatment, and biosensing. In oncology, 2D Por-MOFs serve as efficient photosensitizers for photodynamic therapy (PDT) by generating reactive oxygen species (ROS) to eradicate tumor cells, while also enabling synergistic therapeutic outcomes through integration with chemodynamic therapy (CDT), chemotherapy, immunotherapy, sonodynamic therapy (SDT), and novel mechanisms such as copper-dependent cell death. For antibacterial applications, these materials enhance ROS production via size engineering, single-atom modification, or nanozyme loading, effectively killing pathogens and promoting wound healing, as well as being incorporated into smart dressings to achieve combined hemostatic and antimicrobial functions. In biosensing, 2D Por-MOFs act as ideal platforms for photoelectrochemical signal transduction or fluorescent probes, facilitating the development of highly sensitive fiber-optic SPR, electrochemical, and fluorescence sensors capable of detecting disease biomarkers, pathogens, small-molecule metabolites, and ions with high sensitivity. Finally, the current challenges and future prospects for the clinical translation of 2D Por-MOFs are discussed.

## 1 Introduction

Metal-Organic Frameworks, constructed from metal ions or clusters and organic ligands via coordination bonds, are crystalline porous materials renowned for their tunable structures, high surface areas, and exceptional porosity, showing great promise in gas storage, separation, and catalysis (Furukawa et al., 2013; Zhou and Kitagawa, 2014; Peng et al., 2023; Jin et al., 2024). In recent years, 3D and 2D nanoscale MOFs (nMOFs) have garnered significant attention in biomedicine as versatile nanoplatforms for efficient therapeutics (Sultan et al., 2019; Wen et al., 2021; Sun Z. et al., 2023). Among them, 2D nMOFs-particularly those constructed from porphyrins and their derivatives-have emerged as a powerful class of theranostic platforms (Luo T. et al., 2022; Wang et al., 2025; Huang et al., 2026a). These 2D porphyrin-based MOFs (Por-MOFs) typically exhibit a layered architecture with well-defined thicknesses, which can be categorized into several distinct morphologies: monolayer or few-layer nanosheets (thickness < 5 nm), ultrathin nanosheets (5–20 nm), and nanomembranes (∼1–3 nm) (Luo T. et al., 2022; Li J. et al., 2024; Chen Z. et al., 2022). This unique structure not only retains the functionalizability characteristic of their 3D counterparts but also confers several distinct advantages (Luo T. et al., 2022). Their large surface-to-volume ratio exposes abundant active sites, while their minimal thickness shortens the diffusion path for photogenerated charge carriers, thereby enhancing charge separation efficiency (Xue et al., 2023; Pan et al., 2025). Additionally, the planar geometry of 2D nanomaterials enables close interaction with cell membranes, facilitating improved cellular uptake (Mao et al., 2014; Tu et al., 2018; Chen et al., 2019; Zhang W. et al., 2020; Wang et al., 2024a). Crucially, the direct integration of porphyrin photosensitizers into the MOF backbone effectively suppresses aggregation-induced quenching in physiological environments, thereby preserving high reactive oxygen species (ROS) generation efficiency (Yu et al., 2020; Xu D. et al., 2023; Kuang et al., 2024; Chen H. et al., 2025). This structural design enables superior performance in anticancer and antibacterial applications (Schlachter et al., 2021; Zhang et al., 2024a; Zhang Q. et al., 2024; Qi et al., 2025).

Their exceptional photophysical properties render them particularly well-suited for cancer therapy, especially in PDT (Jiao et al., 2025; Gao et al., 2022; Hu et al., 2025). PDT uses light-activated photosensitizers to generate ROS, which oxidize biomolecules in cancer cells and kill them. Porphyrin-based photosensitizers are among the most widely studied photoactive materials in recent years. Combining them with 2D carriers offers clear advantages. The 2D nature of Por-MOFs significantly enhances therapeutic efficacy by increasing collisions between photosensitizers and molecular O_2_ and by facilitating efficient diffusion of ^1^O_2_-often outperforming their 3D MOF counterparts by more than an order of magnitude (Luo T. et al., 2022). In addition to cancers with high mortality rates, infections caused by pathogenic microorganisms and the emergence of drug-resistant bacteria also pose significant threats to human health (Wang Z. et al., 2022; Xie et al., 2023; Chen C. et al., 2024; Hao et al., 2024; Ralhan et al., 2024). In the field of materials science, a variety of advanced functional materials have demonstrated exceptional antimicrobial properties, finding broad applications in biomedicine, wound dressings, and environmental disinfection (Song et al., 2024; Yan et al., 2025; Long et al., 2022; Zhao et al., 2022). Among them, hydrogels (Chai et al., 2023; Jia et al., 2023; Tang et al., 2023; Yang Y. et al., 2024), nanozymes (Liu Y. et al., 2022; Hou and Xianyu, 2023; Shan et al., 2023; Zhong et al., 2023; Liu L. et al., 2024), polyphenols (Zheng et al., 2022; Liu et al., 2023; Nassarawa et al., 2023; Chen Q. et al., 2024), and MOFs have garnered significant attention due to their tunable structures, high surface area-to-volume ratios, and excellent biocompatibility (Han et al., 2022; Yan et al., 2022). Of particular note, 2D materials have emerged as highly promising candidates for antimicrobial applications owing to their unique physicochemical properties-including ultrahigh specific surface area, tunable surface functionalization, excellent electrical conductivity, and strong interfacial interactions enabled by atomic-scale thickness (Li B. et al., 2022). In terms of antimicrobial mechanisms, various therapeutic strategies-including chemotherapy, metal-ion therapy, gas therapy, CDT, and phototherapy-have been extensively explored for antibacterial applications (Hu et al., 2022; Ma Y. et al., 2022; Wang T.-Y. et al., 2023; Chen L. et al., 2024; He et al., 2024; Zhang F. et al., 2024). Among these, phototherapy has garnered particular attention due to its low cost, ease of implementation, and minimal invasiveness (Kong et al., 2023; Sun J. et al., 2023; Zhao et al., 2023; Li et al., 2024a; Xu et al., 2024; Fakouri et al., 2025; Song et al., 2025). In the face of rising antibiotic resistance, 2D Por-MOFs have also shown strong potential in antimicrobial applications due to their potent ROS generation, where their 2D morphology enhances bacterial membrane contact and damage (Xue et al., 2023). Strategies such as size reduction to expose more active sites, single-atom modification to boost charge separation (He et al., 2025), or hybridization with Ag NPs for combined ROS generation and ion release have significantly improved their antibacterial efficiency against bacteria (Tan et al., 2022).

Accurate detection of diverse biological analytes-ranging from small molecules to macromolecules-is critical for advancing human health and diagnostics (Du et al., 2021; Zhang S. et al., 2021; Zhao et al., 2021). In recent years, surface plasmon resonance (SPR)-based biosensing has attracted considerable interest; however, its limited sensitivity has hindered broader application in biological assays (Hoa et al., 2007; Abbas et al., 2011; Nangare and Patil, 2021; Wang Q. et al., 2022; Ravindran et al., 2023; Tan et al., 2023; Dai et al., 2025). MOFs are innovative hybrid materials offering unique capabilities in biosensing through electrochemical and optical signal transduction for analyte detection (Keum et al., 2023; Mohanty et al., 2024; Saboorizadeh et al., 2024; Ibrahim et al., 2025; Dou et al., 2021). The integration of SPR with MOFs offers a promising strategy to enhance both sensitivity and signal amplification, thereby overcoming current performance barriers. Beyond therapy, 2D Por-MOFs are also excellent candidates for high-performance biosensors, owing to their superior optoelectronic properties and large surface area (Zhang G. et al., 2022; Ma J. et al., 2022). Their conjugated porphyrin framework acts as an efficient photoelectrochemical transducer for enhanced detecting sensitivity (Wang Y. et al., 2022).

**FIGURE 1:**
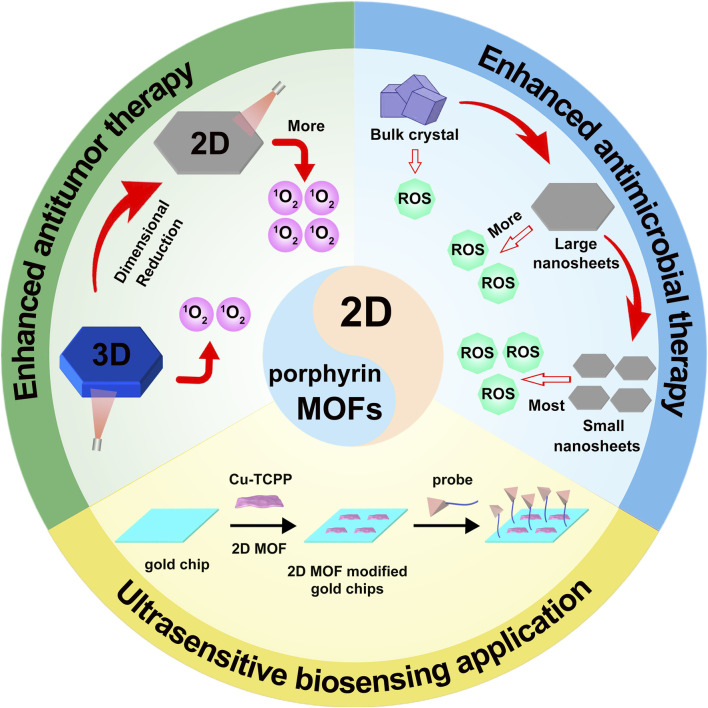
Schematic illustration of 2D Por-MOFs for cancer therapy, antimicrobial applications, and biosensing.

Porphyrin-based MOFs have garnered significant attention as biomedical materials, with several reviews highlighting their promising applications in cancer therapy (Huang et al., 2026a; Akbari Oryani et al., 2025; Tao et al., 2026). This review systematically highlights recent advances in their applications across three key areas: (1) cancer therapy, focusing on enhanced PDT, combination strategies, and intelligent responses to the TME (Section 3); This section demonstrates, through comparative validation, that 2D Por-MOFs exhibit superior anticancer performance due to their enhanced ROS generation (Figure 1). (2) antibacterial applications, including performance optimization through structural engineering and development of smart wound dressings (Section 4); This section highlights the advantages of transitioning from bulk MOFs to 2D MOFs in antibacterial applications (Figure 1). (3) biosensing, covering design principles and applications in SPR, electrochemical, and fluorescence sensing (Section 5). This section will highlight the advantages of 2D Por-MOF-based composites in enhancing sensor performance (Figure 1). Finally, we address key challenges, scalable synthesis, long-term biosafety, and metabolic fate, and outline future directions for clinical translation and development, aiming to advance this smart material toward precision medicine.

## 2 Advantages of 2D Por-MOFs platform for biomedical applications

### 2.1 Diverse synthesis routes enable structural versatility

**FIGURE 2:**
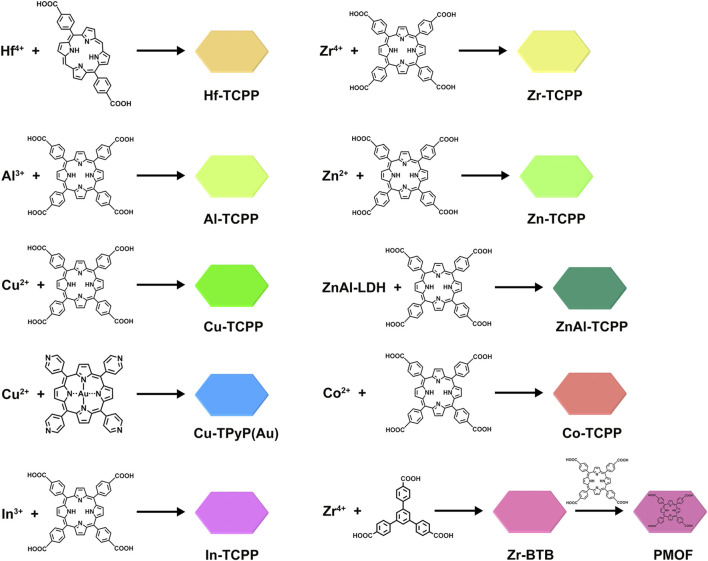
Synthetic procedures for preparing various 2D Por-MOFs using different metals and porphyrin ligands.

As crystalline materials constructed from interconnected metal ions and organic ligands, 2D MOFs combine the advantageous properties of both inorganic and organic components, endowing them with significant potential for diverse biomedical applications (Yang et al., 2022; Wang Y. et al., 2023; Jin et al., 2025; Patil et al., 2025; Wu et al., 2025; Yücer et al., 2025). The synthesis of 2D MOFs can be achieved through a variety of methods, such as solvothermal, exfoliation method, vapor diffusion, liquid-liquid diffusion, direct mixing, ultrasonication, microwave-assisted, and secondary growth techniques (Jian et al., 2020; Liu Y.-L. et al., 2022; Wang L. et al., 2022; Liu J. et al., 2024; Wang et al., 2024b; Wu P. et al., 2024; Xie et al., 2024). This synthetic versatility not only facilitates precise control over morphology and thickness but also enables the incorporation of a broad range of metal centers-from tetravalent Hf^4+^ and Zr^4+^ to divalent Zn^2+^, Cu^2+^, and Co^2+^-as illustrated in Figure 2, which highlights ten representative 2D MOFs explored in biological contexts. Such structural diversity underpins their adaptability and functionality, driving extensive research and development in critical areas including cancer therapy, antibacterial treatment, and biosensing.

Diverse metal centers and varied porphyrin ligands give porphyrin-based MOFs rich structural features. Their porosity directly affects mass transport of O_2_ and substrates to the photosensitizer. High surface area and ordered pores promote ROS generation and diffusion, enhancing PDT efficacy. Metal nodes, such as Cu^2+^, not only tune the electronic structure of the porphyrin ring but also enable enzyme-like catalytic activity, which can synergistically support antibacterial action or modulate the tumor microenvironment. Functionalizing ligands with targeting groups (e.g., folic acid) improves selective recognition and cellular uptake, increasing therapeutic precision. Some modified ligands even impart stimuli-responsiveness (e.g., to pH or GSH), allowing controlled ROS release. Together, these structural elements determine the overall performance of porphyrin-based MOFs in anticancer, antibacterial, and biosensing applications.

### 2.2 Advantages of 2D Por-MOFs for therapeutic applications

This review focuses on 2D Por-MOFs due to their distinct advantages, particularly as carriers in anticancer and antibacterial applications. While porphyrins are widely studied photosensitizing molecules, their inherent hydrophobicity often limits bioavailability and therapeutic efficiency, making the choice of delivery platform critical (Kyropoulou et al., 2020; Li M. et al., 2022; Akbar et al., 2023; Deng C. et al., 2023; Liu S. et al., 2024; Zhang R. et al., 2024; Zhang et al., 2024b). Compared to conventional carriers such as polymeric materials (Chang et al., 2016; Tong et al., 2016; Song et al., 2022; Ulloa Rojas et al., 2022), liposomes (Cressey et al., 2022; Krivosheeva et al., 2023; Liu D. et al., 2024; Yang B. et al., 2024; Alhoussein et al., 2025; Li et al., 2025), covalent organic frameworks (COFs) (Khan et al., 2023; Chen P. et al., 2024; Li X.-G. et al., 2024; Tong S. et al., 2024; Feng et al., 2025; Zhang et al., 2026), or even three-dimensional (3D) MOFs, 2D Por-MOFs offer superior performance by overcoming key limitations-including low drug loading, poor dispersibility, limited functionalizability, and, most importantly, the inability to fully prevent porphyrin aggregation, which severely compromises ROS generation and overall efficacy.

2D Por-MOFs not only enable high porphyrin loading but also utilize their periodic, crystalline architecture to spatially isolate porphyrin units, thereby completely suppressing self-aggregation and maintaining optimal photochemical activity. Furthermore, their large specific surface area and intrinsic porous structure provide ample sites for multifunctional engineering, while the synergistic interplay between metal nodes and organic linkers endows them with unique electronic properties that support diverse activation mechanisms for combination therapy. Notably, compared to their 3D counterparts, 2D Por-MOFs exhibit improved solubility and enhanced accessibility to oxygen molecules owing to their ultrathin morphology (Luo T. et al., 2022). This structural advantage leads to significantly higher ROS production efficiency at lower doses, offering a more effective therapeutic profile (Luo T. et al., 2022).

### 2.3 Advantages of 2D Por-MOFs for biosensing applications

Extensive research has demonstrated that 2D nanomaterials possess unique advantages in the field of biosensing (Lian et al., 2022; Li et al., 2023; Ram and Kumar, 2024; Wang et al., 2024c; Sun et al., 2025). Among these, the ultrathin 2D morphology and exceptionally high specific surface area of 2D Por-MOFs provide a physical foundation for achieving ultra-sensitive detection, while their chemically tunable surfaces enable precise functionalization for specific targeting. The integration of 2D Por-MOFs with surface plasmon resonance (SPR) sensors can significantly enhance sensitivity, response kinetics, and selectivity through multiple synergistic mechanisms (Gao et al., 2024). Specifically, the high electrical conductivity and efficient charge transport capability of 2D MOFs strengthen the localized electromagnetic field at the sensing interface, thereby amplifying the SPR signal (Gao et al., 2024). Concurrently, the intrinsic photosensitivity of MOFs improves photon harvesting efficiency, directly boosting the optical output (Wang Y. et al., 2022). Moreover, their long-range ordered layered crystalline structure facilitates strong coupling between surface plasmons and the gold film’s SPR mode, leading to further signal amplification. The large surface area of the nanosheets also offers abundant binding sites for target molecules, enhancing analyte enrichment. Additionally, the conjugated π-system within the porphyrin ligands enables selective recognition and anchoring of conjugated carbon-based biomolecules-such as DNA and proteins-via π-π stacking interactions, which contributes to an intensified and more specific signal response (Wang G. et al., 2023). Together, these advantages establish a highly efficient and sensitive optical sensing platform.

## 3 2D Por-MOFs for enhanced antitumor therapy

2D Por-MOFs have been extensively studied and widely recognized for their applications in cancer therapy. Their advantages in PDT, in particular, are significantly more pronounced compared to their 3D counterparts (Luo T. et al., 2022). The 2D architecture not only facilitates enhanced oxygen diffusion and efficient release of ROS, but also promotes closer interaction with intracellular biomolecules, thereby maximizing the therapeutic efficacy of PDT. Beyond PDT, 2D Por-MOFs demonstrate distinct benefits across multiple treatment modalities, including SDT, CDT, copper-dependent cell death (cuproptosis), and chemotherapy. As summarized in Table 1, these materials have been successfully applied in diverse anticancer strategies, leveraging their unique structural and functional properties. Collectively, this broad spectrum of therapeutic applications underscores the pivotal role and high research value of 2D Por-MOFs in advancing modern cancer treatment.

**TABLE 1:** 2D Por-MOFs for cancer therapy.

Material	Treatment/ Advantages		Property	Ref
	Treatment	Advantages		
3D Hf-MOF 2D Hf-MOL	PDT/^1^O_2_	Dimensional reduction	Nanoplate, diameter ≈ 200 nm, thickness ≈ 1.7 nm, 630 nm, 4T1 and CT26	Luo T. et al. (2022)
PMOF@HA	PDT/^1^O_2_	Pt NPs for O_2_ generation	Lamellar structure, thickness ≈ 11.65 nm, −17.4 mV, 670 nm, SMMC-7721	Li et al. (2024a)
Cu-TCPP(Al)-Pt-FA	PTT/ Immunotherapy	O_2_ generation, GSH depletion	nanosheets, diameter ≈ 200 nm, 8.2 mV, 638 nm, M109 and HepG2	Chen Z. et al. (2022)
BrP@MOL	PDT/^1^O_2_	tumor oxygenation	Nanoplate, diameter ≈ 150 nm, −4.55 ± 0.19 mV, 630 nm, 4T1 and CT26	Lin et al. (2024)
Zn-TCPP MOF	PDT/^1^O_2_	Acid-responsive	nanosheets, diameter ≈ 90 ± 25 nm, zeta = −5.3 mV, 660 nm, 4T1	Hang et al. (2021)
Fe3O4@Cu-TCPP	PDT/CDT	Independent of O_2_	nanosheet, length ≈ 250 nm, thickness ≈ 2.5 nm, 660 nm, MR Imaging, 4T1	Jiao et al. (2025)
Cu2O/Cu-TCPP/(Pt-Au)/FA	CDT/starvation therapy	O_2_ generation	nanosheet, −26 mV, Russell mechanism, MCF-7, HepG-2, HCMEC-D3	Gao et al. (2022)
ZnAl-TCPP	SDT/PDT	phase transformation for enhanced SPDT	nanosheet, lateral dimensions ≈ 80–120 nm, thickness ≈ 11.1–11.7 nm, 650 nm, ultrasound, 4T1	Hu et al. (2025)
Al-TCPP	SDT	^1^O_2_/·OH	nanosheet, size ≈ 160–200 nm, thickness ≈ 18.4–26.5 nm, ultrasound, 4T1	Zhou et al. (2023)
Cu-T@MH	CDT	GSH depletion, pH/H_2_O_2_-responsive	Rectangle like shape, length ≈ 65 nm, width ≈ 55 nm, thickness ≈ 3 nm, FL imaging, 4T1	Xu et al. (2022)
TAT-AuTPyP-Cu	Cuproptosis/CDT	GSH depletion, O_2_ generation	nanosheet, diameter ≈ 268 nm, thickness≈3 nm, FL/MR Imaging, HeLa	Bao et al. (2024)

Abbreviations: MR (magnetic resonance); FL (fluorescence); SPDT (Sono-photodynamic therapy).

### 3.1 Enhanced PDT efficacy from 3D MOFs to 2D MOFs

**FIGURE 3:**
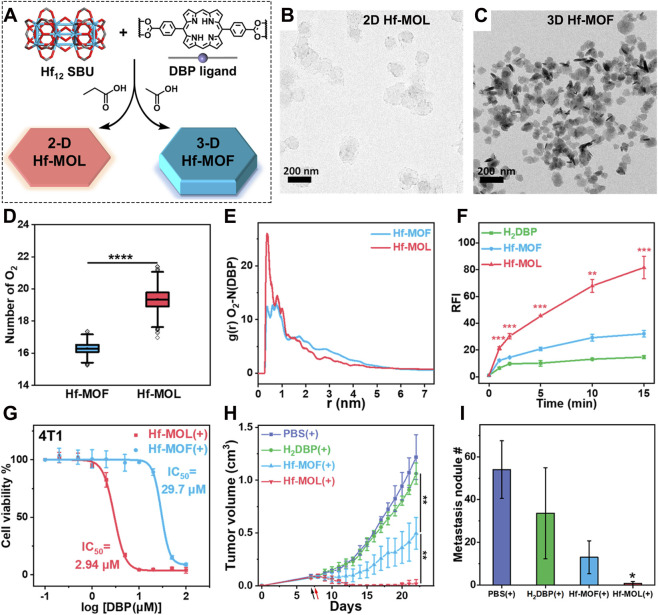
**(A)** Synthetic procedures for 2D Hf-MOL and 3D Hf-MOF. **(B)** TEM image of 2D Hf-MOL. **(C)** TEM image of 3D Hf-MOF. **(D)** Statistical analysis of the number of oxygen molecules near the porphyrin ligands in Hf-MOF and Hf-MOL. **(E)** Radial distribution functions of O_2_ around the DBP nitrogen atoms. **(F)** ^1^O_2_ generation capacity measured by SOSG assay. **(G)** Cytotoxicity of the two materials against 4T1 cells. **(H)** Tumor volume changes in different experimental groups. **(I)** Number of metastatic nodules after treatment with different experimental groups. Reproduced with permission from (Luo T. et al., 2022). Copyright (2022), American Chemical Society.

To demonstrate the phototherapeutic superiority of 2D over 3D structures, Luo et al. synthesized both 2D Hf-MOL and 3D Hf-MOF using Hf^4+^ and the DBP ligand, systematically investigating how dimensionality affects the photodynamic performance of Por-MOFs (Figure 3A) (Luo T. et al., 2022). TEM images in Figures 3B,C confirmed the successful preparation of the two distinct morphologies. Molecular dynamics (MD) simulations further revealed that oxygen molecules were more densely distributed around the DBP photosensitizers in the Hf-MOL, particularly in the lateral direction (Figure 3D), with a higher radial distribution density compared to the 3D MOF (Figure 3E). Consistently, ^1^O_2_ generation assays showed significantly enhanced production in the 2D Hf-DBP (Figure 3F). In biological evaluation, the 2D Hf-MOL exhibited dramatically improved cytotoxicity against 4T1 cancer cells under light irradiation, with an IC_50_ of 2.94 μM-over tenfold lower than that of the 3D Hf-MOF (29.7 μM) (Figure 3G). *In vivo* studies further demonstrated that the 2D Hf-MOL achieved superior suppression of tumor growth (Figure 3H) and metastasis (Figure 3I), highlighting the significant therapeutic advantage conferred by the 2D architecture. This dimensional engineering strategy clearly validates the enhanced therapeutic potential and promising application prospects of 2D MOFs.

### 3.2 Overcoming hypoxia and enabling smart responsiveness for enhanced PDT

**FIGURE 4:**
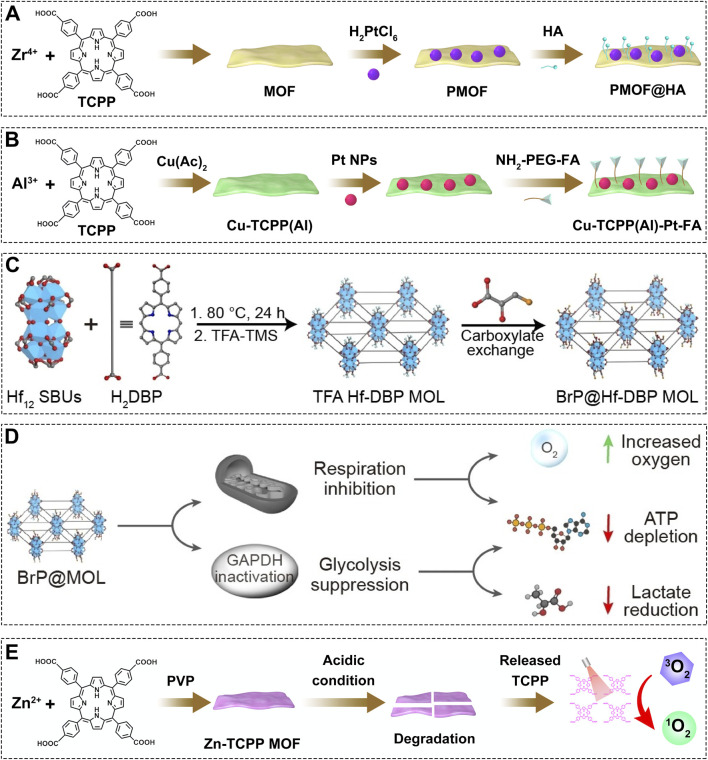
**(A)** Preparation procedure of the PMOF@HA composite. **(B)** Synthesis steps of the Cu-TCPP(Al)-Pt-FA composite. **(C)** Synthesis steps of the BrP@Hf-DBP MOL composite. **(D)** Mechanism of BrP@MOL for increasing local oxygen concentration. Reproduced with permission from (Lin et al., 2024). Copyright (2024), Wiley-VCH GmbH. **(E)** Schematic illustration of Zn-TCPP MOF degradation under acidic conditions and subsequent PDT effect.

While the intrinsic advantages of the 2D structure provide a solid foundation for developing efficient PDT platforms, the complex tumor microenvironment (TME)-particularly its inherent hypoxia and high levels of reducing agents like GSH-remains a major barrier to achieving optimal PDT outcomes (Gong et al., 2025; Wu M.-Y. et al., 2024; Diao et al., 2025; Jia et al., 2025; Xu C. et al., 2025; Zhang Z. et al., 2020; Lan et al., 2021; Cheng et al., 2024; Chen L. et al., 2025; Xu T. et al., 2025). Therefore, simply possessing a favorable 2D morphology is insufficient; there is a pressing need to design intelligent, multifunctional nanoplatforms capable of actively remodeling this hostile microenvironment to fully unlock the therapeutic potential of 2D materials. To address hypoxia, Yao et al. developed a platinum nanoparticle-decorated 2D Zr-TCPP MOF (PMOF@HA) (Figure 4A), where the Pt NPs exhibit catalase-like activity to convert endogenous H_2_O_2_ into O_2_, thereby alleviating oxygen deficiency and boosting PDT efficacy (Li J. et al., 2024). In parallel, to counteract GSH-mediated ROS scavenging, Chen et al. engineered a hybrid Al-based 2D MOF (Al-TCPP), followed by Cu coordination to the porphyrin core and subsequent deposition of Pt nanoparticles and folic acid (FA) modification, yielding Cu-TCPP(Al)-Pt-FA (Figure 4B) (Chen Z. et al., 2022). Upon targeted delivery to cancer cells, the Pt component generates O_2_ from H_2_O_2_, while the Cu sites selectively deplete GSH, synergistically enhancing PDT through dual microenvironment modulation. Beyond oxygen generation, an alternative “oxygen-saving” strategy involves metabolic reprogramming by inhibiting mitochondrial respiration (Zheng et al., 2021; Luo G. et al., 2022; Zhang H. et al., 2022; Cen et al., 2023; Tian et al., 2023; Yu et al., 2023; Zhong et al., 2024). For instance, Lin et al. constructed a 2D Hf-based MOL loaded with 3-bromopyruvate (3-BrP), a dual inhibitor of glycolysis and mitochondrial respiration (Figure 4C) (Lin et al., 2024). After cellular internalization, BrP@Hf-DBP MOL reduces tumor oxygen consumption by suppressing energy metabolism, thereby improving oxygen availability and enhancing PDT (Figure 4D). Furthermore, leveraging the unique acidic pH of the TME, Jiang et al. designed a Zn-TCPP MOF (Figure 4E) that remains relatively inactive under neutral conditions but releases free TCPP molecules in the weakly acidic tumor environment, leading to a significant increase in ^1^O_2_ generation (Hang et al., 2021). This pH-responsive activation offers a novel paradigm for designing smart, tumor-selective photosensitizers.

### 3.3 Synergistic therapy combining PDT with other modalities

While remodeling the tumor microenvironment has significantly enhanced the efficacy of standalone PDT, the heterogeneity and complexity of cancer often limit the outcomes of monotherapy and promote therapeutic resistance (Chen et al., 2020; Yang et al., 2021). To achieve more potent and comprehensive tumor eradication, research has naturally evolved from “enhancing single modalities” to “integrating multiple synergistic therapies” (Qi et al., 2024; Zou et al., 2024; Song et al., 2021; Li et al., 2022a; Deng J. et al., 2023; Lakkakula et al., 2024; Li et al., 2024b; Gu et al., 2025). Research on combination therapy has demonstrated its significant therapeutic efficacy (Zhao et al., 2020; Xu Y. et al., 2023; Zhang L. et al., 2024; Liu et al., 2025; Weng et al., 2025). Leveraging the facile functionalizability of 2D MOFs, researchers have successfully combined PDT with CDT, SDT, chemotherapy, and other approaches, achieving a synergistic “1 + 1 > 2” effect.

**FIGURE 5:**
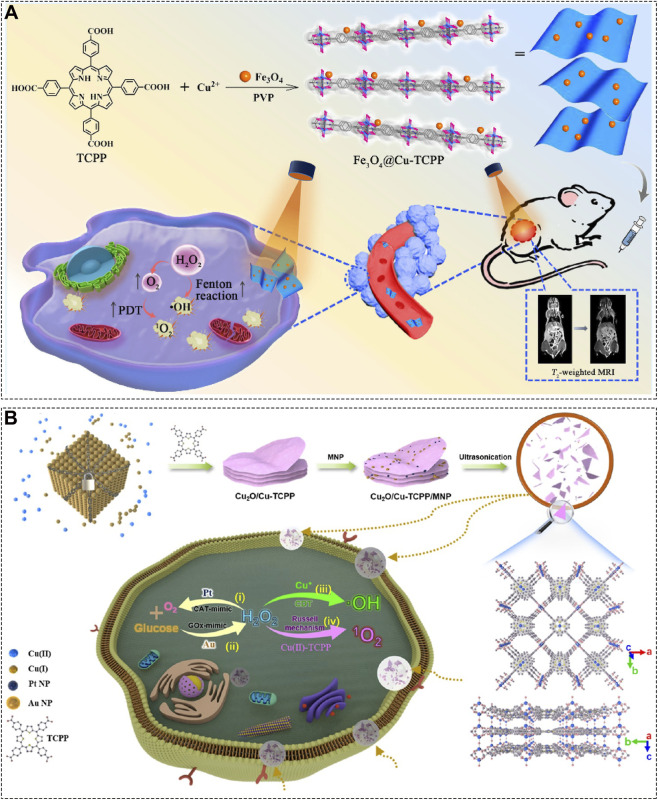
**(A)** Synthesis procedure and anticancer mechanism of Fe_3_O_4_@Cu-TCPP. Reproduced with permission from (Jiao et al., 2025). Copyright (2025), American Chemical Society. **(B)** Preparation steps and anticancer schematic of Cu_2_O/Cu-TCPP/MNP. Reproduced with permission from (Gao et al., 2022). Copyright (2022), American Chemical Society.

For instance, Jiao et al. constructed a 2D Cu^2+^-based MOF nanosheet loaded with Fe_3_O_4_ nanoparticles and modified with polyvinylpyrrolidone (PVP), yielding the Fe_3_O_4_@Cu-TCPP composite (Figure 5A) (Jiao et al., 2025). Under light irradiation, the Cu-TCPP nanosheet simultaneously generates ^1^O_2_ for PDT and catalyzes hydroxyl radical (•OH) production via CDT. Notably, the photogenerated electrons from Cu-TCPP facilitate ligand-to-metal charge transfer, which enhances the Fenton reaction of Fe_3_O_4_, thereby boosting •OH generation. The small amount of O_2_ produced in this process also helps alleviate hypoxia, further improving the PDT efficacy of Cu-TCPP. This smart design exemplifies how 2D MOF carriers can enable self-activated, oxygen-independent synergistic PDT/CDT.

The unique structural features of 2D MOF nanosheets further allow for the integration of multiple metal species and therapeutic functions. Gao et al., for example, used Cu_2_O as both a copper source and template to synthesize Cu_2_O/Cu-TCPP, then decorated it with Pt and Au nanoparticles to create the multifunctional Cu_2_O/Cu-TCPP/(Pt-Au) platform (Figure 5B) (Gao et al., 2022). In this system, Cu-TCPP produces ^1^O_2_ via the Russell mechanism under acidic conditions in the presence of H_2_O_2_. Cu^+^ ions from Cu_2_O drives •OH generation through a Fenton-like reaction. Pt nanoparticles catalyze O_2_ production from H_2_O_2_ to relieve hypoxia; while Au nanoparticles consume glucose to disrupt energy supply, enabling starvation therapy. This elegantly engineered nanoplatform not only highlights the versatility of 2D MOFs as carriers but also presents a promising candidate for multimodal cancer treatment.

### 3.4 SDT, CDT, and emerging cell death pathways

**FIGURE 6:**
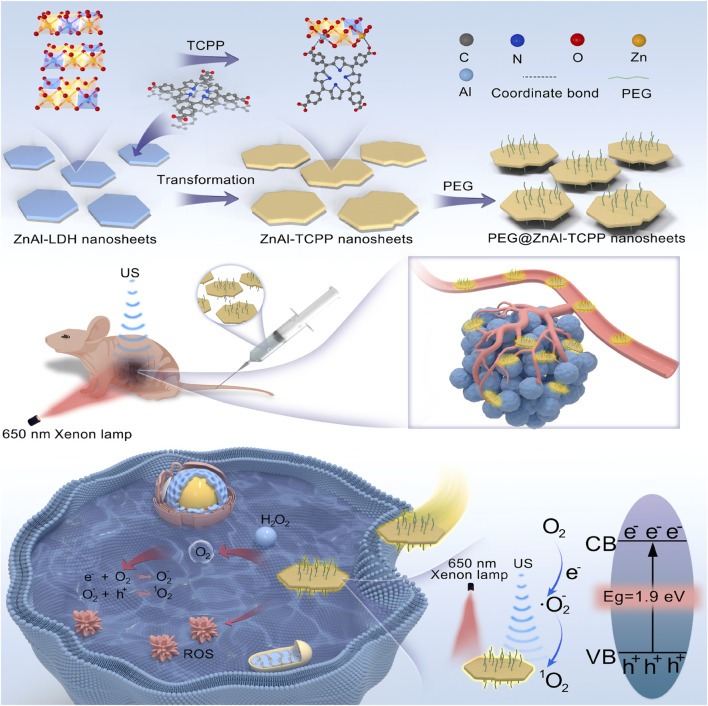
Synthesis procedure and anticancer mechanism of PEG@ZnAl-TCPP under ultrasound activation. Reproduced with permission from (Hu et al., 2025). Copyright (2025), Elsevier.

While PDT and its synergistic strategies show great promise for superficial tumors, limited tissue penetration of light remains a critical barrier for treating deep-seated solid tumors. To overcome this challenge, research has shifted toward alternative stimuli with greater penetration depth, among which ultrasound has emerged as an ideal candidate (Geng et al., 2024; Huang et al., 2026b), giving rise to SDT and opening a new avenue for utilizing 2D Por-MOFs in deep-tumor therapy. Liang et al., for example, employed a phase-transition strategy using 2D ZnAl-layered double hydroxide (LDH) nanosheets as precursors to synthesize 2D ZnAl-TCPP nanosheets, which were further PEGylated to form PEG@ZnAl-TCPP (Figure 6) (Hu et al., 2025). This composite efficiently generated ROS under both light and ultrasound, outperforming either single activation mode and exhibiting a threefold increase in ROS yield compared to free TCPP molecules. Mechanistic studies attributed this enhancement to the phase transformation from 2D LDH to 2D MOF, which significantly boosted the ROS-generating capability of the TCPP photosensitizer. This PDT-SDT combination offers a novel paradigm for expanding the biomedical applications of 2D MOFs.

**FIGURE 7:**
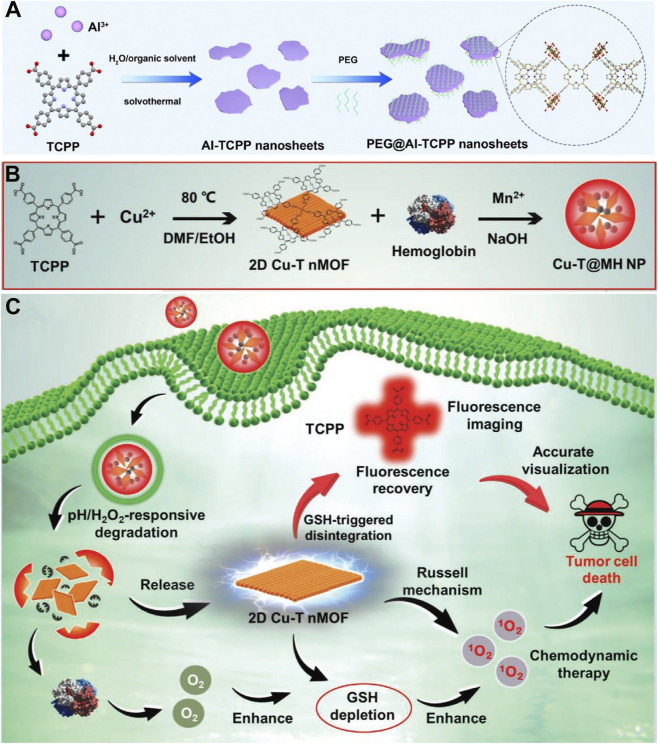
**(A)** Preparation procedure of PEG@Al-TCPP nanosheets. Reproduced with permission from (Zhou et al., 2023). Copyright (2023), The Royal Society of Chemistry and the Chinese Chemical Society. **(B)** Synthesis steps of Cu-T@MH NPs and **(C)** schematic illustration of their multimodal anticancer mechanisms. Reproduced with permission from (Xu et al., 2022). Copyright (2022), Wiley-VCH GmbH.

Although SDT overcomes the depth limitation of light, the efficacy of ROS-based therapies is still constrained by the high antioxidant capacity (e.g., GSH) within tumor cells. Cutting-edge research is now moving beyond traditional ROS-induced apoptosis toward strategies that directly deplete cellular antioxidants or induce novel, highly efficient forms of regulated cell death (RCD), such as cuproptosis-a fundamental shift from “external attack” to “internal disruption.” Tan et al. developed 2D Al-TCPP nanosheets (Figure 7A), demonstrating their ability to efficiently produce both ^1^O_2_ and •OH under ultrasound, showcasing excellent sonodynamic antitumor activity (Zhou et al., 2023). Xu et al. employed biomineralization to encapsulate 2D Cu-TCPP with hemoglobin and MnO_2_, forming the Cu-T@MH nanoparticles (Figure 7B) (Xu et al., 2022). In the acidic, H_2_O_2_-rich tumor environment, Cu-TCPP is released and reacts with H_2_O_2_ to generate ^1^O_2_. Meanwhile, hemoglobin delivers O_2_, and MnO_2_ consumes H_2_O_2_ to produce additional O_2_, facilitating the oxidation of Cu^+^ to Cu^2+^ and thereby depleting intracellular GSH, which strengthens CDT (Figure 7C).

**FIGURE 8:**
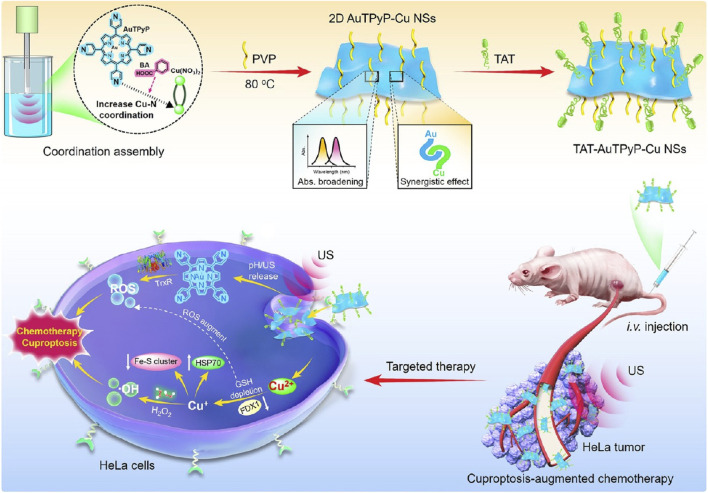
Preparation procedure and antitumor schematic of the TAT-AuTPyP-Cu NSs composite. Reproduced with permission from (Bao et al., 2024). Copyright (2024), American Chemical Society.

The redox cycling between Cu^2+^ and GSH to form Cu^+^ not only fuels the Fenton reaction for •OH generation and reduces ROS scavenging but also activates the Ferredoxin 1-mediated cuproptosis pathway. Bai et al. synthesized a 2D MOF from tetrakis (4-pyridyl)porphyrin and Cu^2+^, then surface-modified it with a cell-penetrating peptide (TAT) to obtain TAT-AuTPyP-Cu nanosheets (Figure 8) (Bao et al., 2024). These nanosheets accumulate efficiently in tumors and, upon cellular uptake and ultrasound activation, release pyridylporphyrin and Cu^2+^ ions, triggering combined cuproptosis and CDT. Additionally, the Au centers in the chelated porphyrin react with overexpressed thioredoxin reductase in cancer cells to generate massive ROS, leading to effective tumor cell death. This work provides valuable insights into the structure-function relationship of 2D Por-MOFs and offers a blueprint for highly efficient tumor ablation.

## 4 2D Por-MOFs for enhanced antimicrobial therapy

In recent years, research on nanomaterials for antimicrobial therapy has emerged as a prominent focus due to their unique physicochemical properties and potent antimicrobial efficacy, demonstrating significant scientific value and promising applications in addressing critical public health challenges such as drug-resistant bacterial infections (Wang et al., 2021; Zhang W. et al., 2021; Lu et al., 2022; Wang X. et al., 2023). While 2D Por-MOFs have demonstrated remarkable synergistic potential in combating internal threats such as cancer, their applicability extends far beyond oncology. Excitingly, the core design principle-utilizing enhanced ROS generation to precisely destroy target cells-has been successfully adapted to address external biological threats, particularly pathogenic microbial infections. Strategies refined in anticancer research, such as minimizing particle size to expose more active sites or incorporating platinum single atoms (Pt SAs) to optimize charge separation efficiency, have been directly leveraged to significantly boost photodynamic antimicrobial performance. This indicates that a nanoplatform validated in the tumor microenvironment can be ingeniously repurposed into a powerful weapon against drug-resistant bacteria. More importantly, their application form has evolved from systemic administration in cancer therapy to localized delivery in antimicrobial applications-for instance, by integrating with GelMA to fabricate smart aerogel patches that combine hemostatic and photodynamic antibacterial functions. This transformation marks a shift from a single therapeutic agent toward multifunctional biomedical materials, offering a novel solution to the global crisis of antibiotic resistance.

### 4.1 From 3D to 2D and size control for performance enhancement

**FIGURE 9:**
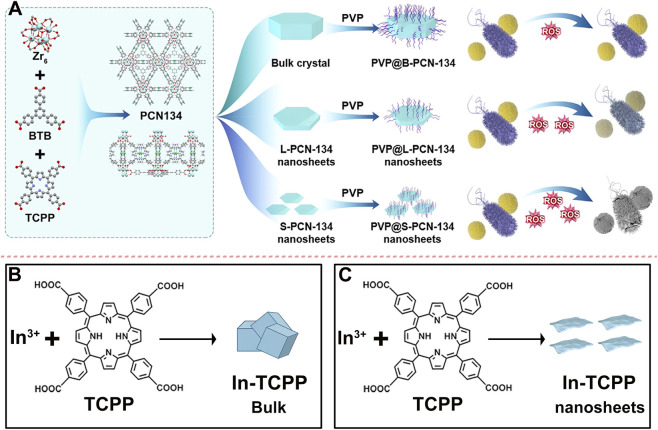
**(A)** Preparation procedures of porphyrinic MOF materials with three different morphologies and schematic illustration of their antibacterial efficiency. Reproduced with permission from (Xue et al., 2023). Copyright (2023), Elsevier. **(B)** Synthesis steps of In-TCPP Bulk and **(C)** In-TCPP nanosheets.

Compared to bulk or 3D MOFs, 2D MOFs exhibit significantly enhanced photodynamic activity, which contributes to superior antimicrobial efficacy. Literature evidence consistently highlights the advantages of 2D MOFs in this domain. For example, Zhao et al. synthesized three morphologies of MOF materials-3D crystals, large-sized 2D nanosheets, and small-sized 2D nanosheets-and formulated them into PVP-based composites (Figure 9A) (Xue et al., 2023). They found that the small-sized 2D MOF, possessing the highest specific surface area, displayed the strongest photodynamic antibacterial activity (Figure 9A). This systematic comparative study directly demonstrates that reducing MOF dimensionality-from bulk 3D structures to 2D nanosheets, and further to smaller nanosheets-dramatically enhances photodynamic activity (ROS yield) and antimicrobial performance, providing compelling evidence for the “dimensional advantage” emphasized in this review. In another study, Pan et al. prepared both 3D and 2D MOFs based on In^3+^ and TCPP (Figures 9B,C) (Pan et al., 2025). The resulting 2D In^3+^-MOF nanosheets exhibited enhanced PDT antibacterial performance compared to their bulk counterparts, attributed to a higher exposure of active sites. This finding further validates, from a different material system, the universality and effectiveness of the proposed “2D strategy.”

### 4.2 Single-atom and nanoparticle decoration for synergy

The above studies establish the critical role of the 2D structure itself in boosting photodynamic antimicrobial activity. However, to push performance boundaries and address complex infection environments, researchers have moved beyond intrinsic optimization and instead focus on precise functionalization of 2D MOF nanosheets to introduce new active sites and functionalities. By integrating single atoms or nanoparticles, it becomes possible to modulate electronic structures or introduce multiple antimicrobial mechanisms, thereby achieving synergistic enhancement.

**FIGURE 10:**
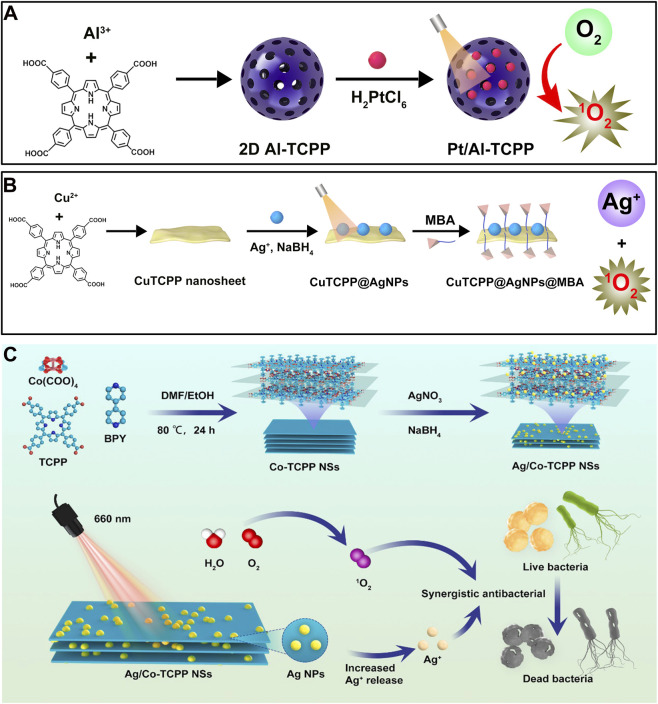
**(A)** Synthesis procedure of Pt/Al-TCPP. **(B)** Synthesis steps of the multifunctional Pt/Al-TCPP. **(C)** Synthesis steps of Ag/Co-TCPP NSs and schematic illustration of their antibacterial mechanism. Reproduced with permission from (Li et al., 2022b). Copyright (2022), Elsevier.

To further amplify the photodynamic activity of 2D MOFs for optimal efficacy under low drug concentrations and mild light irradiation, recent work has explored the incorporation of metal elements that synergize with the MOF’s porous crystalline framework to improve light harvesting. For instance, He et al. fabricated 2D Al-TCPP MOFs and loaded them with Pt single atoms (SAs) (Figure 10A) (He et al., 2025). This fine-tuned modification leverages atomic-level engineering-where Pt SAs optimize the electronic structure and promote charge separation-to intrinsically enhance the photodynamic capability of the MOF nanosheets, representing an “atomic-scale” boosting strategy that enables effective wound healing under low-dose irradiation. Metal nanoparticles themselves possess inherent antimicrobial properties; combining them with biocidal metals like Ag can significantly strengthen the antibacterial effect. Yang et al., for example, developed a 2D Cu-TCPP MOF decorated with Ag nanoparticles and functionalized with mercaptophenylboronic acid (MBA) for targeted recognition (Figure 10B) (Tan et al., 2022). Benefiting from the synergistic action of Ag nanoparticles and MBA-mediated targeting, the CuTCPP@AgNPs@MBA system exhibited potent bactericidal activity against *Staphylococcus aureus*. This “functional integration” approach-combining ROS generation, Ag^+^ release, and targeted binding-enhances antibacterial efficacy and underscores the high tunability and robust anti-infection potential of 2D MOFs. Similarly, Zhou et al. constructed Ag nanoparticle-decorated 2D MOFs (Ag/Co-TCPP NSs) (Figure 10C), where the combination of Ag NPs and PDT led to synergistic bacterial killing (Li et al., 2022b).

### 4.3 Application integration and innovation from therapeutic agent to multifunctional medical device

**FIGURE 11:**
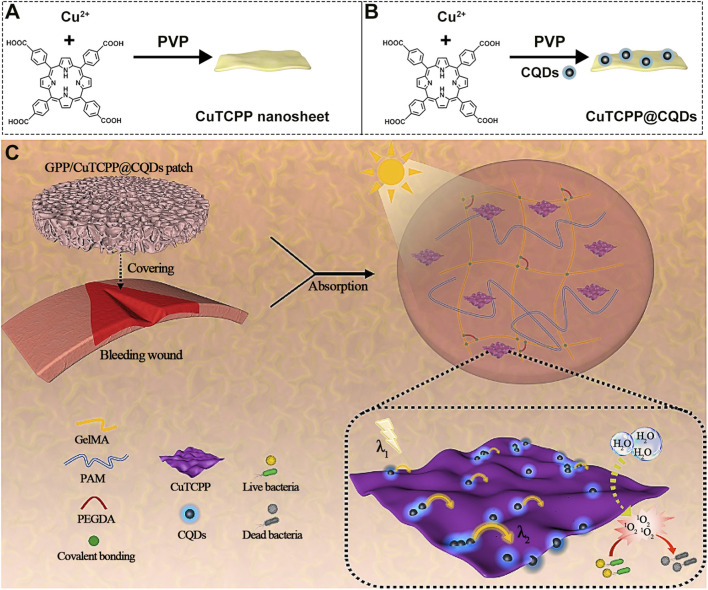
**(A)** Synthesis procedure of CuTCPP nanosheet and **(B)** CuTCPP@CQDs. **(C)** Schematic illustration of the antibacterial mechanism of the GPP/CuTCPP@CQDs patch. Reproduced with permission from (Wang et al., 2024d). Copyright (2024), Elsevier.

Antibacterial performance of 2D MOF-based nanotherapeutics has been greatly enhanced through atomic- and nano-level modifications. However, translating these high-performing laboratory results into practical clinical applications requires addressing challenges related to delivery methods, retention at infection sites, and the integration of additional therapeutic functions. The latest research frontier is focused on integrating these advanced nanotherapeutics with biomaterials to create user-friendly, functionally integrated medical devices, thus advancing to the next stage of application. For example, Wei et al. developed quantum dot- and PVP-modified 2D MOF materials (Figures 11A,B) (Wang et al., 2024d). In this work, they incorporated CQD-modified 2D Cu-based MOF nanosheets as the active component into a multifunctional aerogel patch, achieving dual hemostatic and antibacterial capabilities-representing a significant leap from “nanodrug” to “applicable medical device” (Figure 11C). Importantly, this study exemplifies a key translational pathway for 2D MOF-based antimicrobial materials: integration with biomaterials to construct novel medical devices with real clinical potential. It transcends the simple paradigm of “killing bacteria” and addresses two critical clinical needs in wound management-hemostasis and infection control-greatly enhancing the practical value and clinical relevance of 2D MOFs.

## 5 2D Por-MOFs for biosensing applications

The exceptional performance of 2D Por-MOFs in antimicrobial applications fundamentally stems from their efficient interaction with biological interfaces (e.g., bacterial membranes) and robust ROS generation. These same characteristics-particularly their outstanding photoelectronic properties and large surface area-also lay a solid foundation for their application in another cutting-edge field: high-sensitivity biosensing. However, their role here shifts from an active “therapeutic warrior” to a sensitive “diagnostic sentinel.” Instead of using ROS to destroy targets, they now leverage their superior optoelectronic properties, such as high electron mobility, and vast surface area to capture biological recognition events and transduce them into readable electrical or optical signals. Studies have shown that integrating 2D Por-MOFs with surface plasmon resonance (SPR) sensors significantly enhances three key sensor parameters, attributable to five synergistic mechanisms: first, the high conductivity and charge mobility of 2D MOFs strengthen the excitation field and amplify surface plasmon resonance at the sensing interface; second, the intrinsic photoelectric properties of MOFs enhance light absorption, directly boosting signal output; third, the long-range ordered layered crystalline structure promotes coupling between surface plasmon waves and the SPR mode of the gold film; fourth, the large surface area of the 2D nanosheets provides abundant active sites for analyte binding; and fifth, the conjugated π-system of the porphyrin ligand enables strong interaction with conjugated carbon-based biomolecules via π-π stacking, facilitating the capture and enrichment of signal molecules.

### 5.1 SPR enhancement and fluorescent probes for optical sensing

**FIGURE 12:**
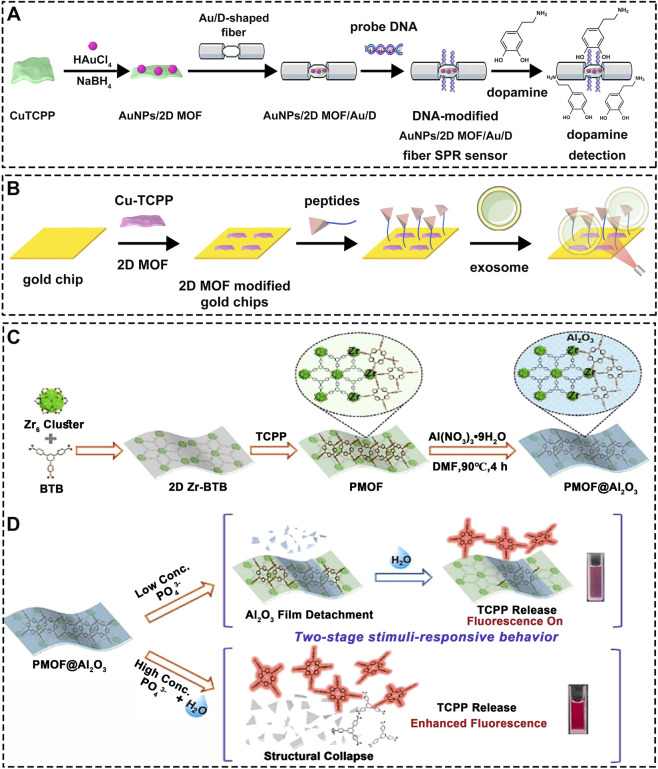
**(A)** Schematic illustration of the fabrication process for the SPR sensor by integrating CuTCPP with AuNPs and its detection mechanism for dopamine. **(B)** Detection mechanism of the sensor prepared by combining Cu-TCPP with a gold chip for exosome analysis. **(C)** Synthesis procedure of PMOF@Al_2_O_3_ and **(D)** its mechanism for phosphate ion detection. Reproduced with permission from (Zhang G. et al., 2022). Copyright (2022), Elsevier.

2D MOFs play a crucial role in enhancing the performance of optical sensors, serving either as a sensitive signal amplifier or as an intelligent responsive core. For instance, Jiang et al. designed a 2D MOF platform loaded with Au nanoparticles and integrated it with a D-shaped fiber to achieve a two-level amplification of the SPR signal (Figure 12A) (Gao et al., 2024). By further coupling this platform with a DNA probe, they achieved highly sensitive detection of dopamine molecules in both PBS and serum solutions. This system exemplifies the application of 2D MOFs in SPR sensors, where the coupling between 2D Cu-TCPP MOF and gold nanostructures creates a strong electromagnetic field, enhancing SPR sensitivity for efficient detection of small biomolecules. Beyond small molecules, 2D Cu-TCPP MOF can also act directly as an SPR enhancer for large biomolecules. Chen et al., for example, immobilized 2D Cu-TCPP MOF onto a gold chip and combined it with functional peptides to enable efficient detection of exosomes (Figure 12B) (Wang Y. et al., 2022). By leveraging the MOF’s excellent conductivity, enhanced light absorption, and large surface area, this system significantly improved the sensitivity and efficiency of conventional SPR sensors for detecting cancer biomarkers like exosomes. In addition to organic analytes, the detection of inorganic ions such as phosphate-an essential nutrient in biological systems-is also critical. To this end, Chai et al. synthesized a 2D Zr-BTB MOF and utilized the unsaturated Zr^4+^ sites to chelate TCPP molecules, followed by coating with an Al_2_O_3_ shell to form the PMOF@Al_2_O_3_ composite (Figure 12C) (Zhang G. et al., 2022). They found that low concentrations of phosphate could selectively disrupt the Al_2_O_3_ layer and bind to Zr^4+^, triggering the release of fluorescent TCPP molecules for detection (Figure 12D). At higher concentrations, phosphate not only induced fluorescence but also completely disintegrated the 2D MOF (Figure 12D). This elegantly designed stimulus-responsive fluorescent probe demonstrates the potential of 2D MOFs in constructing smart, highly selective sensors for inorganic ions.

### 5.2 Enzymatic and non-enzymatic platforms for electrochemical sensing

**FIGURE 13:**
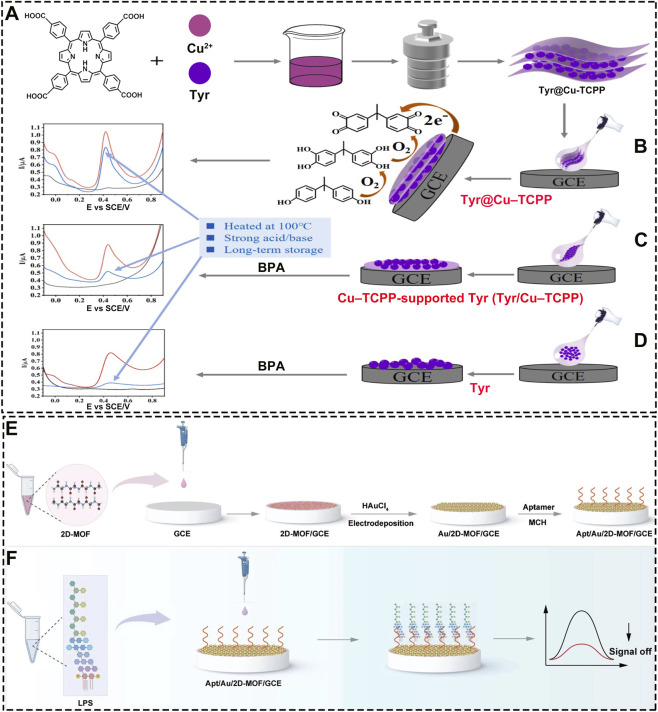
**(A)** Synthesis procedure of the Tyr@Cu-TCPP composite and stability comparison of Tyr@Cu-TCPP **(B)**, Cu-TCPP loaded with tyrosinase (Tyr/Cu-TCPP, **(C)**, and free tyrosinase (Tyr, **(D)** for electrochemical bisphenol A (BPA) biosensing. Reproduced with permission from (Ma J. et al., 2022). Copyright (2022), Elsevier. **(E)** Preparation steps of the Apt/Au/2D-MOF/GCE composite. **(F)** Signal response of the Apt/Au/2D-MOF/GCE composite for LPS detection. Reproduced with permission from (Tong Y. et al., 2024). Copyright (2024), Springer Vienna.

The outstanding optical properties of 2D MOF materials have enabled remarkable advances in optical sensing. However, for applications requiring even higher sensitivity, point-of-care testing, or specific enzymatic reactions, electrochemical sensing offers distinct advantages. Benefiting from their tunable conductivity and abundant active sites, 2D MOFs have emerged as ideal materials for constructing next-generation electrochemical platforms, enabling a transition from optical to electrochemical detection modalities. Biosensing technology has gained widespread recognition among researchers for its application in food safety and has emerged as a crucial tool for the real-time and precise detection of hazardous substances in food (Dou et al., 2021). Enzyme-based electrochemical sensors hold significant promise in food safety. Zheng et al., for example, integrated tyrosinase (Tyr) with a 2D Por-MOF to form a nanocomposite membrane (Tyr@Cu-TCPP) (Figure 13A), which served as an ultrasensitive electrochemical biosensor for bisphenol A detection (Ma J. et al., 2022). Compared to free tyrosinase or enzyme simply loaded onto Cu-TCPP, the assembled Tyr@Cu-TCPP membrane exhibited superior stability (Figures 13B–D), particularly under high-temperature conditions where natural enzymes typically denature. This study presents a paradigm for enzyme-based electrochemical sensors, where the 2D MOF (Cu-TCPP) acts as a “nanocage” to stabilize enzyme molecules, significantly enhancing both stability and performance-effectively addressing the long-standing challenge of thermal instability in biosensors. Beyond enzymatic platforms, non-enzymatic sensors have also shown great potential for biomolecule detection. Dai et al., for instance, developed a non-enzymatic electrochemical sensor by combining gold nanoparticles with a 2D MOF, demonstrating that Cu-TCPP MOF serves as an excellent electrode modifier for constructing an aptamer-based sensor to effectively detect lipopolysaccharide (LPS) (Figures 13E,F) (Tong Y. et al., 2024). Collectively, these studies underscore the significant value of 2D MOFs in advancing electrochemical sensing technologies.

### 5.3 Multifunctional applications from cellular imaging to single-cell analysis

**FIGURE 14:**
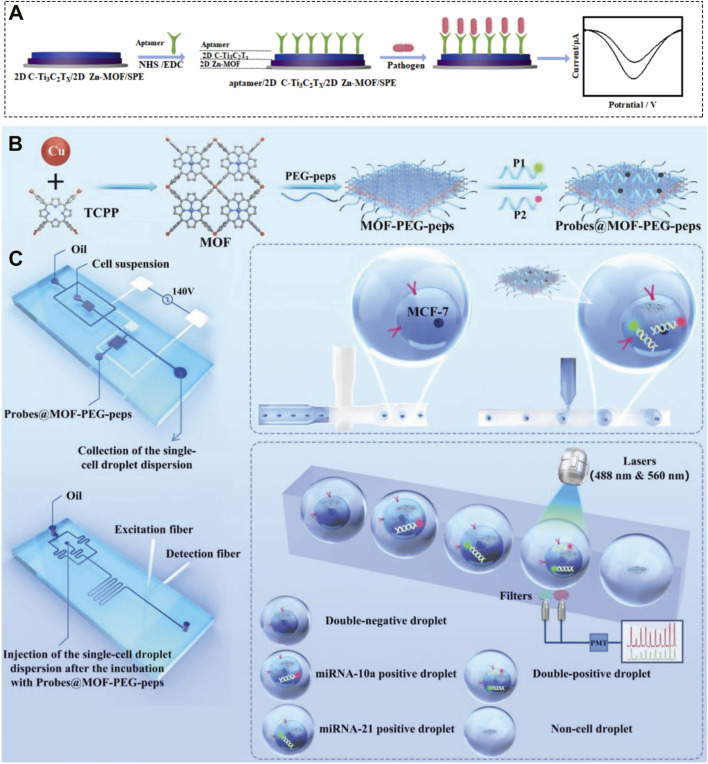
**(A)** Schematic illustration of a 2D MOF integrated with aptamers and 2D carboxylated C-Ti_3_C_2_T_X_ for detection of *E. coli* and *S. aureus*. Reproduced with permission from (Yang et al., 2025). Copyright (2025), Elsevier. **(B)** Synthesis procedure of the Probes@MOF-PEG-peps composite and **(C)** schematic illustration of its application as sensors for *in situ* detection of intracellular miRNA. Reproduced with permission from (Chen J. et al., 2022). Copyright (2022), Wiley-VCH GmbH.

Whether in optical or electrochemical sensing, the ultimate goal is the precise and efficient detection of target analytes to address real-world biomedical challenges. Moving beyond single-function detection, the latest research is focused on developing 2D MOF-based sensors as multifunctional integrated platforms for more demanding applications, such as intracellular imaging, rapid screening of complex samples, and even unprecedented single-cell analysis. For example, Jiao et al. employed 2D Cu-MOF nanosheets as a carrier to load DNA probes, enabling high-sensitivity tracking and co-localization analysis of circRNA-miRNA interactions within living cells (Wang G. et al., 2023). This work highlights the carrier capability of 2D MOFs combined with DNA cascade amplification, significantly improving detection sensitivity and demonstrating their broad potential in medical biosensing. Beyond RNA analysis in living cells, 2D MOFs can also be used to detect pathogens. Mo et al. constructed a multifunctional electrochemical sensor by integrating a 2D MOF with aptamers and 2D carboxylated C-Ti_3_C_2_T_X_, enabling effective detection of *E. coli* and *S. aureus* (Figure 14A) (Yang et al., 2025). This integrated design showcases the potential of 2D MOFs in developing portable and efficient point-of-care testing (POCT) devices.

Furthermore, 2D MOFs can be engineered into sensors for *in situ* detection of intracellular miRNA. Yang et al., for instance, functionalized 2D Cu-TCPP nanosheets with dual-color fluorescently labeled DNA probes to create a fluorescence resonance energy transfer (FRET) nanosensor (Figure 14B), achieving simultaneous detection of two miRNAs in breast cancer cells (Figure 14C) (Chen J. et al., 2022). By combining 2D MOF nanoprobes with digital droplet microfluidics-a cutting-edge technology-they realized multiplexed miRNA detection within single circulating tumor cells (Figure 14C), representing the frontier of ultra-sensitive and high-resolution biosensing. This work provides a blueprint for developing non-invasive tools for early detection of cancer metastasis.

Through the exploration of biosensing applications, this review highlights how 2D Por-MOFs, with their precise “sensing” capabilities, offer powerful tools for early diagnosis and real-time monitoring. Their detection ability spans multiple biological scales-from resolving circRNA-miRNA regulatory axes at the single-cell level to ultrasensitive detection of LPS in serum-covering nearly every level from micro to macro. This powerful diagnostic capability forms a perfect complement and closed loop with their robust “executing” functions in anticancer and antimicrobial therapies. An ideal future scenario would involve using a 2D MOF-based sensor for early detection of cancer biomarkers or pathogen infections, followed by precise intervention using a 2D MOF-based therapeutic platform, with simultaneous imaging for treatment monitoring-ultimately achieving true theranostics. Together, these three pillars-precise diagnosis, efficient therapy, and real-time monitoring-sketch a complete blueprint for precision medicine, fully demonstrating the immense value and boundless potential of 2D Por-MOFs as a new generation of intelligent biomedical platforms.

## 6 Conclusion

This review systematically highlights the significant potential of 2D Por-MOFs in three major biomedical fields: anticancer therapy, antimicrobial treatment, and biosensing. Leveraging their unique ultrathin 2D morphology, exceptionally high surface area, tunable porosity, outstanding optoelectronic properties, and the ability to immobilize a high density of photosensitizers in a spatially isolated manner, these materials effectively overcome the inherent limitations of conventional small-molecule photosensitizers-such as aggregation-caused quenching, poor stability, and limited multifunctionality. In cancer therapy, they serve not only as highly efficient PDT agents but also as versatile platforms integrating CDT, chemotherapy, immunomodulation, and even emerging modalities like “cuproptosis.” For antimicrobial applications, through size engineering, single-atom modification, and nanocomposite formation, they achieve dramatically enhanced photodynamic efficacy and have been successfully integrated into smart dressings, demonstrating promising prospects for combined hemostatic and antibacterial functions. In biosensing, their superior photoelectrochemical characteristics and abundant functionalization sites make them ideal building blocks for constructing highly sensitive SPR, electrochemical, and fluorescent sensors, enabling ultrasensitive detection of targets ranging from disease biomarkers to environmental pollutants. Collectively, these advancements underscore the role of 2D Por-MOFs as an “all-in-one” theranostic platform, showcasing their transformative significance and disruptive potential in advancing precision medicine.

Although 2D Por-MOFs show great promise in biomedical applications, such as cancer therapy, antibacterial treatment, and biosensing, their clinical translation still faces several common challenges. First, systematic evaluation of their *in vivo* biocompatibility and long-term toxicity remains insufficient. While most studies report good cytocompatibility *in vitro*, the *in vivo* degradation behavior and potential accumulation toxicity of metal ions (e.g., Cu^2+^, Hf^4+^) are not yet well understood. Second, performance stability in complex biological environments is limited. For example, poor penetration into hypoxic tumor cores, blocking of active sites by proteins or biofilms in infected wounds, and non-specific adsorption in serum samples all hinder reliable function. Third, current synthesis methods are mostly confined to lab-scale batches. They struggle to meet the stringent requirements for pharmaceutical or diagnostic use, particularly regarding purity, batch-to-batch consistency, and reproducibility.

Future progress should focus on three integrated strategies: smart material design, precise environmental response, and practical engineering integration. At the material level, researchers should prioritize endogenous metals like Fe or Zn. Alternatively, they can develop “smart” MOFs that respond selectively to disease-specific cues, such as acidic pH or high GSH in tumors, or specific enzymes and mild acidity at infection sites, to enable controlled activation and reduce off-target effects. At the functional level, multimodal approaches offer significant advantages. Combining PTT, PDT, and metal-ion release can enhance efficacy against heterogeneous tumors or drug-resistant bacteria. Integrating imaging modalities (fluorescence/MRI) further allows real-time tracking of drug delivery and therapeutic response. At the application level, engineering practical formulations is essential. 2D Por-MOFs should be incorporated into user-friendly formats, such as hydrogels, patches, or microfluidic chips, for wound care or point-of-care testing. Surface modification with anti-fouling layers (PEG or zwitterionic polymers) can minimize non-specific binding. Direct growth of MOFs on electrode fibers or embedding them in stable polymer matrices also improves mechanical robustness and signal reproducibility. Together, these efforts will help bridge the gap between promising lab-scale prototypes and real-world clinical or field-deployable solutions. Moreover, to advance clinical translation, collaboration with materials manufacturing companies is needed to standardize production processes. Partnerships with biopharmaceutical companies are also essential to thoroughly evaluate the *in vivo* metabolism of these materials.

In summary, 2D porphyrinic MOFs stand as a brilliant bridge connecting materials science and biomedicine. Their future advancement will undoubtedly depend on close interdisciplinary collaboration among chemists, materials scientists, biologists, and clinicians. By deeply understanding their structure-property relationships, rationally designing intelligent functionalities, and systematically addressing the challenges related to safety, scalability, and practical implementation, we have every reason to believe that these remarkable materials will bring revolutionary advances to human health and disease diagnosis and therapy.
